# The modified pancreatic stent system for prevention of post-ERCP pancreatitis: a case-control study

**DOI:** 10.1186/s12876-017-0661-2

**Published:** 2017-10-18

**Authors:** Cheng Zhang, Yu-long Yang, Yue-feng Ma, Hong-wei Zhang, Jing-yi Li, Mei-ju Lin, Li-jun Shi, Chun-chun Qi

**Affiliations:** Department of Biliary Minimally Invasive Surgery, Affiliated Zhongshan Hospital of Dalian University, No, 6. Jiefang Road, Zhongshan District, Dalian, Liaoning Province 116001 People’s Republic of China

**Keywords:** Acute pancreatitis, Endoscopic retrograde cholangio pancreatography, Pancreatic stent, Endoscopic nasal biliary drainage

## Abstract

**Background:**

Prophylactic pancreatic stents after endoscopic retrograde cholangiopancreatography (ERCP) can help prevent post-ERCP pancreatitis. However most of the pancreatic stents need to be removed by another ERCP. The aim of this observational study was to investigate the feasibility and effectiveness of the modified pancreatic stent system for prevention of post-ERCP pancreatitis.

**Methods:**

From November 2013 to November 2015, a total of 230 patients who had prophylactic pancreatic stent placed for prevention of post-ERCP pancreatitis at a single institution were identified and stratified. In this case-control design, 150 patients received an ordinary pancreatic stent, and 80 patients received the modified pancreatic stent. The main outcome measures were the difficulty level and complications of pancreatic stent placement and extraction between the two groups.

**Results:**

In ordinary group, the average time of pancreatic stent and nasal biliary drainage placement was 3.5 ± 0.6 min. There were 13 cases of stent proximal migration (8.7%), 20 cases of stent spontaneous abscission (13.3%), 5 cases of acute pancreatitis (3.3%) (2 cases for stent abscission) and 7 cases of hyperamylasemia (4.7%) after ERCP. One hundred thirty patients received extra duodenoscope (86.7%) to remove the stent, and 4 cases had acute pancreatitis and 5 patients had hyperamylasemia after removing the proximal migratory stents. In modified group, the average time of pancreatic stent system placement was 4.9 ± 0.7 min, but there was only one case of stent abscission (1.3%), 2 cases of acute pancreatitis (2.5%) and 3 cases of hyperamylasemia (3.8%). The new pancreatic stents were removed directly under x-ray without complication.

**Conclusions:**

The modified pancreatic stent system has the same effect of preventing post-ERCP pancreatitis, lower rate of stents proximal migration and spontaneous abscission, and the advantage of easier removed compared with ordinary pancreatic stent.

## Background

Endoscopic retrograde cholangiopancreatography (ERCP) is the primary method used to manage pancreatobiliary disease, but it is also an invasive procedure that carries significant risks for the patients. The most common complication from the endoscopic sphincterotomy (EST) is acute pancreatitis, and other complications from the procedure include perforations, sepsis and bleeding [1]. Post-ERCP pancreatitis (PEP) is defined as acute abdominal pain within 48 h following ERCP with levels of serum lipase elevated at least 3-fold and a requirement for analgesic drugs for at least 24 h. A systematic survey of 21 prospective studies with 16,855 patients conducted between 1987 and 2003 found a 3.5% occurrence of PEP, 0.4% instances of severe pancreatitis and 0.11% deaths [2].

There are a number of risk factors associated with PEP and they can be divided into either patient-related risk factors or endoscopist-related risk factors. Patient-related risk factors include female gender, previous pancreatitis and Sphincter of Oddi Dysfunction (SOD) [3]. Endoscopist-related risk factors include difficult instrumentation of papilla and pancreatic duct, precut sphincterotomy, endoscopic sphincterotomy, endoscopic pancreatic sphincterotomy (EPT), injection of contrast medium into the pancreatic duct and intraductal ultrasonography [4, 5]. The consequences of these risk factors are various injuries including, mechanical injury, thermal injury, hydrostatic injury, chemical injury, allergic injury, enzymatic injury with intraluminal activation of proteolytic enzymes and infection from contaminated endoscope and accessories.

Despite the introduction of various techniques over several decades to prevent PEP or limit its severity only a few strategies have been proven effective and have been integrated into clinical practice. Several systematic reviews and meta-analyses of randomized, double-blind, clinical trials have examined pancreatic stent placement and the efficacy of drugs, such as diclofenac, somatostatin, and nonsteroidal anti-inflammatory drugs to reduce the incidence of PEP [6–9]. In a meta-analysis of controlled clinical trials involving 481 patients the group that did not have stents implanted had 3-fold higher odds of developing pancreatitis compared with the group of patients that were treated with stents (15.5% vs. 5.8%; Odds Ratio (OR) 3.2: 95% Confidence Interval. Number needed to treat analysis showed that one in every 10 patients could be expected to benefit from pancreatic-duct stent placement [10]. A more recent meta-analysis of 1541 patients found that prophylactic pancreatic stent (PS) placement prevented PEP after ERCP compared with no PS placement thus supporting the importance of PS placement after ERCP for the prevention of PEP [11].

Plastic stents can be divided into three categories including straight PS, single pigtail PS and double pigtail PS. The straight PS and single pigtail PS are commonly used for pancreatic duct drainage. Compared to straight PS single pigtail PS has been demonstrated to minimize stent proximal migration, but there is a higher incidence of spontaneous abscission with single pigtail PS.

The observations made in the previous studies prompted us to design a modified of pancreatic stent system for prevention of PEP. In our system, the PS can be removed along with the nasobiliary catheter. The aim of this observational study was to investigate the feasibility and effectiveness of the modified pancreatic stent system for prevention of post-ERCP pancreatitis.

## Methods

### Design

This study was an analysis of clinical outcomes data associated with different types of pancreatic stent placement. It is a retrospective review of patient medical records documented in the Department of Biliary Minimally Invasive Surgery affiliated to Zhongshan Hospital of Dalian University in China. The study was approved by the Conduct of Human Ethics Committee of the Affiliated Zhongshan Hospital of Dalian University.

### Patients

A single endoscopist performed ERCP in 735 consecutive patients with pancreatobiliary disease from November 2013 to November 2015. Exclusion criteria were, malignant tumor with biliary metal stent insertion, pancreatic duct stone, and cases that did not place nasobiliary drainage tubes at the same time. Two hundred thirty patients who had prophylactic pancreatic stent placed were eligible for inclusion. One hundred fifty received an ordinary pancreatic stent and nasobiliary drainage tubes (ordinary group) from November 2013 to October 2014 and 80 received the modified pancreatic stent (Modified group) from November 2014 to November 2015. The main outcome measures were the difficulty level and complications of pancreatic stent placement and extraction between the two groups.

### Endoscopic equipment and accessories

The following equipment and accessories were utilized during endoscopy: JF-260v/TJF-240 electronic duodenoscope (Olympus, Japan), VIO-200 s high frequency generator (mixed currents, cut current of 40-W, coagulation current of 40-W) (ERBE, German), papillary sphincter knife (Endo-Flex, German), balloon dilatation catheter (balloon diameter: 6 to 12 mm, length: 4 cm, pressure: 8 to 18 ATM) (OptiMed, German), inflation device (Boston Scientific, USA), yellow zebra guide wire, pancreatic stent, nasal biliary drainage tube (Boston Scientific, USA), sutures (Wego, China).

### Standard and method of pancreatic stent placement

Prophylactic PS should be placed if the patient has more than two factors as following: younger age, female gender, previous pancreatitis, SOD, normal serum bilirubin, difficult cannulation, precut sphincterotomy, EST, EPT, pancreatic duct injection, intraductal ultrasonography, sphincter of Oddi manometry, minor papilla sphincterotomy and trainee involvement in procedure [12]. PS was required be placed if the patient was diagnosed with acute or chronic pancreatitis or in patients in which contrast medium in the pancreatic duct drained slowly.

### Therapeutic endoscopy

ERCP was performed using digital subtraction angiography (DSA). EST was performed using a high frequency generator with the following settings: blend 1, cutting of 55, and coagulation of 30. The bile or pancreatic duct was first accessed by insertion of a soft-tipped Teflon tracer (diameter 0.035 in.) guidewire through a 6F, double channel sphincterotome. This was followed by cannulation, injection of contrast solution, EST, and endoscopic papillary balloon dilation (EPBD). Stones were removed by basket or balloon catheter and endoscopic nasal biliary drainage (ENBD) was performed to drain infected bile.

**Fig. 1:**
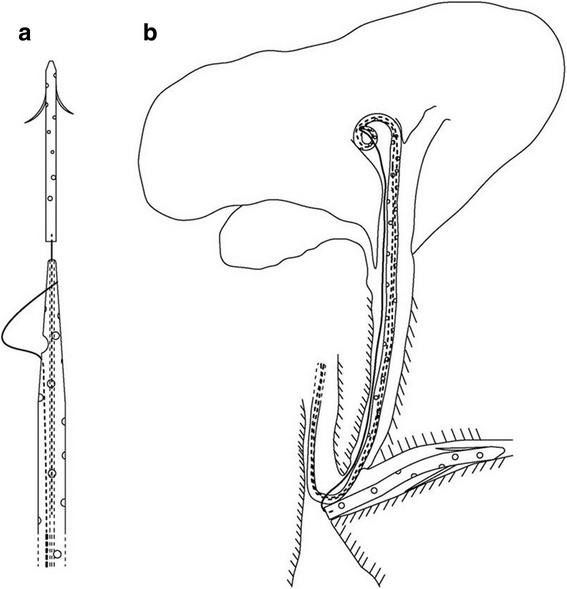
Illustrated diagram showing type three pancreatic stent used in this study: (**a**) The straight pancreatic stent and the nasobiliary catheter is hitched on a common guide line and the nasobiliary catheter takes the place of pancreatic stent propeller. **b** The straight pancreatic stent is placed in the pancreatic duct while the nasobiliary catheter is in common bile duct and two drainage tubes are connected by a line. The line is connected to the end of pancreatic stent and comes out through the head of the nasobiliary catheter. The pancreatic stent can be removed by pulling the nasobiliary catheter and the line

**Fig. 2:**
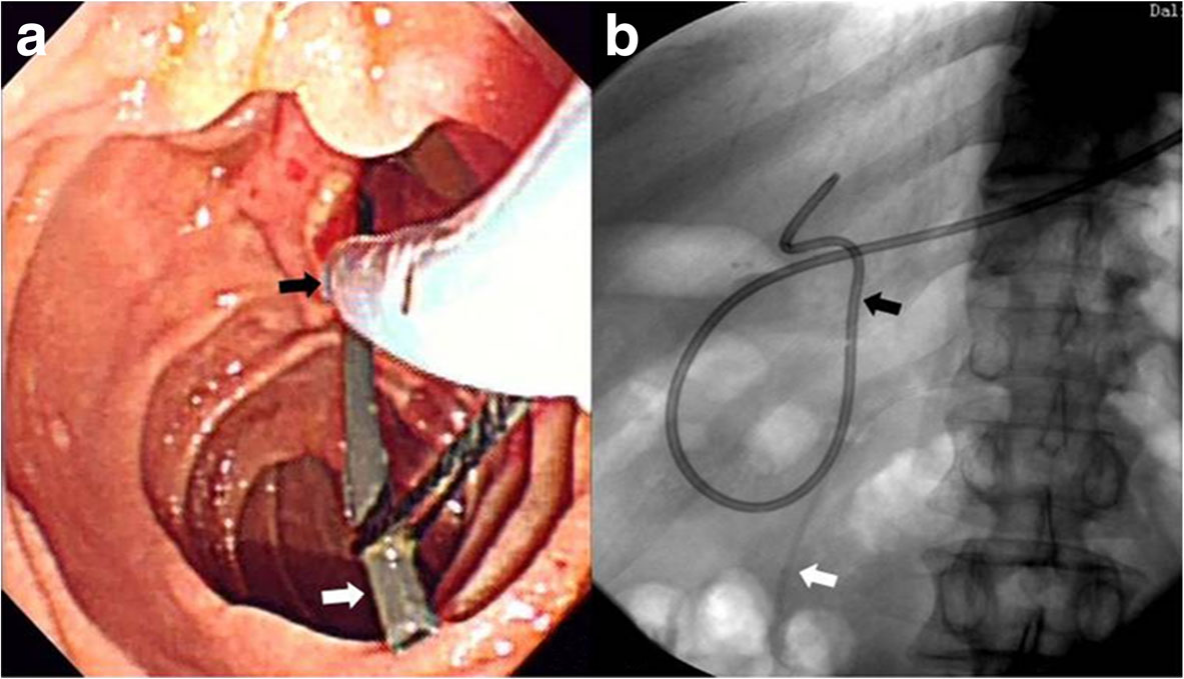
Physical map of type three pancreatic stent: the white arrow indicates the pancreatic stent. The black arrow indicates the nasobiliary catheter. **a** An endoscopic image that shows the pancreatic stent placement in the pancreatic duct. **b** An X-ray image that shows the nasobiliary catheter in common bile duct and the PS linked together by the line

An ordinary PS is pushed into the main pancreatic duct by a PS propeller with guidewire. In modified group (Fig. 1) (Patent number in China: 201,510,238,034.9), the end of the straight PS and the head of the nasobiliary catheter are connected by a line. After the successful placement of PS, nasobiliary catheter will be separated from the PS when the guide wire is pulled back into the lumen of the nasobiliary catheter. Then nasobiliary catheter with guidewire is cannulated into the common bile duct and pushed into the proper position. When the line at end of nasobiliary catheter is stretched two drainage tubes will link together (Fig. 2).

**Fig. 3:**
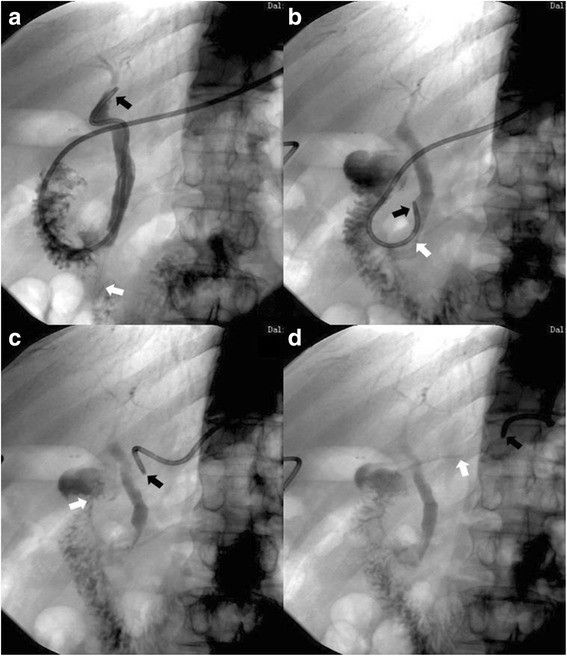
Images illustrating the removal of a pancreatic stent and the nasobiliary catheter type three: The white arrows indicate the end of pancreatic stent and the head of the nasobiliary catheter is indicated by the black arrow. **a** The normal position of pancreatic stent and the nasobiliary catheter. **b** Part of nasobiliary catheter goes into duodenal lumen. **c** The nasobiliary catheter is removed from the common bile duct and part of pancreatic stent is pulled out of pancreatic duct. **d** The entire pancreatic stent is remove from the pancreatic duct

The modified PS system can be removed a week after ERCP (Fig. 3). Cholangiography is performed through nasobiliary catheter to judge whether there is residual stone in common bile duct. To remove the PS the line is tightened and nasobiliary catheter, along with the PS, can be pulled out slowly using X-ray imaging as a guide.

### Determination of clinical outcomes from patient records

Patient records were reviewed to confirm pancreatobiliary disease, efficacy of operation and complications. Complications included, the result of preoperative imaging, intraoperative endoscopy and cholangiography, postoperative laboratory results, X-ray results and laboratory results following the removal of PS. Imaging workup included hepatobiliary pancreas spleen ultrasound, computerized tomography (CT), and magnetic resonance cholangiopancreatography (MRCP). Laboratory workup included blood and urine analysis, as well as serum lipase and liver function tests (LFTs). Endoscopy and cholangiography included the type of nasobiliary catheter and PS. X-ray was used to document the location of stents.

### Data collection

We searched the entire electronic medical record in the Department of Biliary Minimally Invasive Surgery affiliated to Zhongshan Hospital of Dalian University in China. The data of interest recorded in the electronic medical record were collected and tabulated.

### Statistical analysis

Descriptive statistics were used to characterize the quantitative date with means and 95% confidence intervals. The two-tailed student’s t-test was used to assess for differences between continuous means of the two groups. The two-tailed Fisher exact and chisquare tests were used to compare categorical variables. *P* < 0.05 was considered to be statistically significant. All statistical analyses were performed using SPSS software package, version 21 (Statistical Package for Social Sciences, IBM Corporation, Armonk, NY, USA).

## Results

### Patients characteristics and major primary disease

Demographic characteristics of the patients and univariable analyses for the outcome variables are presented in “Table 1”. There was no statistical significance between Ordinary group and modified group (*P* > 0.05).

**Table 1:** Clinical characteristics of the 230 patients

	Ordinary group		New group		*P*
	N	%	N	%	
Gender					
Male	89	59.3	46	57.5	
Female	61	40.7	34	42.5	0.888
Age (Years)	65.9 ± 14.5		62.5 ± 12.3		0.072
Major primary disease					
Choledocholithiasis	87	58.0	50	62.4	0.573
Sphincter stenosis	15	10.0	5	6.3	0.463
SOD	10	6.7	6	7.5	0.792
Periampullary diverticula	18	12.0	11	13.8	0.683
Pancreaticobiliary maljunction	11	7.3	5	6.3	1.000
Duodenal papillitis	9	6.0	3	3.7	0.550

### Time of stent placement and hemodlastase and serum lipase in perioperative period

The average time of modified pancreatic stent system placement was 4.9 ± 0.7 min, and the ordinary pancreatic stent and nasal biliary drainage placement was 3.5 ± 0.6 min. There was statistical significance between two groups (*P* < 0.05). Hemodlastase and serum lipase in perioperative period are presented in “Table 2”. There was no statistical significance between Ordinary group and modified group (*P* > 0.05).

**Table 2:** Hemodlastase and serum lipase of the 214 patients

	Ordinary group	New group	*P*
Placement time (min)	3.5 ± 0.6	4.9 ± 0.7	0.000
Hemodlastase (U/L)			
Preoperative	85.4 ± 48.9	81.9 ± 41.2	0.591
Postoperative	251.2 ± 348.7	235.2 ± 184.9	0.704
pancreatic stent extubation	81.8 ± 42.1	79.3 ± 25.8	0.622
Serum lipase (U/L)			
Preoperative	172.8 ± 216.2	222.1 ± 175.7	0.081
Postoperative	620.8 ± 871.5	643.5 ± 737.4	0.843
pancreatic stent extubation	203.3 ± 192.9	214.7 ± 161.5	0.652

### Effectiveness of complication after ERCP

Review of the records for 230 patients was completed during one session. No perforation, bleeding, heart arrest or other serious complication occurred during stent placement. All patients were able to tolerate the discomfort caused by the nasobiliary drains, and there was no case happened that the nasobiliary drain was removed due to patient’s discomfort or intolerance. However, In the ordinary stent group, there were 13 cases of pancreatic stent proximal migration (8.7%), 20 cases of pancreatic stent spontaneous abscission (13.3%), 5 cases of acute pancreatitis (3.3%) and 7 cases of hyperamylasemia (4.7%) after ERCP operation. In the modified stent group, there was only one case of pancreatic stent abscission observed (1.3%), 2 cases of acute pancreatitis (2.5%) and 3 cases of hyperamylasemia (3.8%). Of the 5 cases, 2 cases had acute pancreatitis for pancreatic stent abscission, and a second ERCP was performed to place modified pancreatic stent. The other patients who were diagnosed with acute pancreatitis and hyperamylasemia recovered following drug treatment. Between ordinary stent group and modified stent group there was no difference in acute pancreatitis (3.3% vs 2.5%, *P* > 0.05) and hyperamylasemia (4.7% vs 3.8%, *p* > 0.05), but there was obvious difference in stent proximal migration (8.7% vs 0%, *p* < 0.05) and stent abscission observed (13.3% vs 1.3%, *p* < 0.05) (Table 3).

**Table 3:** Complication of pancreatic stents placement

	Ordinary group		New group		*P*
	N	%	N	%	
Proximal migration	13	8.7	0	0	0.005
Spontaneous abscission	20	13.3	1	1.3	0.001
Acute pancreatitis	5	3.3	2	2.5	1.000
Hyperamylasemia	7	4.7	3	3.8	1.000

### The removal of pancreatic stent after ERCP

In the ordinary stent group, 130 patients received extra gastroscope or duodenoscope (86.7%) to remove the ordinary pancreatic stents. Two cases had acute pancreatitis and 3 patients had hyperamylasemia after removing the proximal migratory stents. The 5 patients recovered following drug treatment. The new pancreatic stents were removed directly under X-ray and there was no complication happened.

## Discussion

The positioning of the pancreatic stent (PS) across the ampulla and pancreatic sphincter into the pancreatic duct is presumed to maintain the flow of pancreatic secretions across any flow disruptions caused by injury or edema of these structures. Over the past few decades, substantial evidence has supported that 5-7 cm, 5-Fr diameter, plastic, straight PS seems to be safe, efficacious, and resistant to PEP, especially in high-risk patients [13].

Despite the efficacy there are several complications following stent placement including, stent migration or stent occlusion. Several case reports have detailed rare instances of severe complications. There has been a case report in which the PS migrated into the retroperitoneum posterior to the third part of the duodenum, causing duodenal edema and narrowing [14]. One case reported that a PS migrated into the bile duct causing cholangitis [15]. Another case reported that a PS migrated into the portal vein causing portal vein thrombosis [16]. Finally a case reported perforation of the splenic artery after PS placement in chronic obstructing pancreatitis [17].

More commonly, distal and proximal (upstream) migration of pancreatic duct stents may occur with incidence rates of 7.5% and 5.2%, respectively [18]. If there is no acute pancreatitis after ERCP then spontaneous abscission of the PS is beneficial for the patients since the second ERCP can be avoided. A number of meta-analyses of prospective single-center and multi-center studies have been carried out to identify risk factors for post-ERCP pancreatitis. However, it is unclear why patients with these risk factors are at the highest risk for PEP, and these risk factors do not reliably tell us who will have pancreatitis. In our study, 5 cases of spontaneous PS abscission and 3 cases of acute pancreatitis for pancreatic stent abscission were detected. Fortunately, these patients recovered through drug treatment. If medication is contraindicated with aggravated abdominal pain and continuously rising hematuria amylase, urgent salvage ERCP must be performed to replace the PS [19]. In the modified of pancreatic stent tested in this study, the PS was connected with nasobiliary catheter by a line thus spontaneous abscission of PS will not happen. In our study, one PS was removed after the nasobiliary catheter had migrated distal, however, the distal migration incidence rate of nasobiliary catheter was only 2.1%, which is lower then distal migration of a ordinary PS [20].

A long-term pancreatic stent in pancreas duct can predispose patients to chronic pancreatitis, pancreatolith or pancreatic sepsis. Stent retrieval is important to prevent long-term serious ductal damage, but the removal of proximally migrated stent is technically challenging. Basket or balloon technique is the standard method to retrieve proximally migrated pancreatic stents [21]. The lasso technique can also be used and involves inserting a guide wire through the lumen of the migrated stent followed by insertion of a partially opened polypectomy or gooseneck snare over the wire to grasp the stent [22, 23]. Rat-tooth forceps can also used but a small pancreatic duct can preclude full opening of the forceps. Additionally, when the tip of the stent impacts the duct the rat-tooth forceps technique may not be successful. Instead a grasping tripod removal of the stent has been reported to be helpful [24]. There have only been two previous reports on the use of SpyGlass pancreatoscopy to remove migrated pancreatic duct stents. SpyGlass pancreatoscopy facilitates successful guide wire cannulation of migrated stents, which can then be removed with a Soehendra Stent Retriever [25]. We had reported 7 cases of proximally migrated PS with the incidence rates of 2.9%, and these proximally migrated stents were successfully removed by basket and balloon [26]. In the modified pancreatic stent system, proximal migration of PS will be avoided by using a line that is linked with a nasobiliary catheter.

Many endoscopists are trying to get away from the habit of routinely inserted nasobiliary drains, since nasobiliary drain can increase the procedure time and patients’ discomfort, while biliary drainage by nasobiliary drain and drainage by stent are equally safe and effective treatments for acute cholangitis [27–29]. A second endoscopy is needed to remove the biliary stent, which will also increase patient’s discomfort and costs. In China, a nasobiliary catheter placement is recommended after ERCP procedure, since ENBD not only significantly reduces the incidence of hyperamylasemia and pancreatitis but also decreases the length of hospital stay in patients with EST, EPBD and repeated stone extraction [30, 31]. Compared with 6-Fr nasobiliary catheter, the 4-Fr nasobiliary catheter is useful to reduce nasal discomfort [32].

Currently, a PS is mostly occluded as early as 1 month after insertion [33], A follow-up procedure to remove the stent is generally recommended within 1–2 weeks following surgery to avoid the complication of stent occlusion or inward stent migration [34]. A meta-analysis showed that prophylactic pancreatic stents made the probability of pancreatitis dropped to 1.3%, therefore, 98.7% PS could be removed in a week [35]. With the modified pancreatic stent system, a second endoscopy can be avoided because the PS will be removed along with the nasobiliary catheter. The new system greatly reduces the pain of the patients and the costs associated with follow-up procedures. If the line of nasobiliary catheter in type one of the modified pancreatic stent system is released and a guide line is input into the nasobiliary catheter, the loop at the head of the nasobiliary catheter will become straight. Then, the nasobiliary catheter can be removed with the PS remaining in pancreatic duct. In type one of the modified pancreatic stent system this can be achieved if the line is removed.

## Conclusions

In summary, this study investigated the feasibility and effectiveness of a modified pancreatic stent system for prevention of post-ERCP pancreatitis. Our analysis indicated that the modified pancreatic stent system had the same effect of preventing post-ERCP pancreatitis, no obvious difficulty of stent placement, lower rate of stents proximal migration and spontaneous abscission, and the advantage of easier removed compared with ordinary pancreatic stent. Therefore this modified pancreatic stent system could be considered as a complementary medical approach for prevention of post-ERCP pancreatitis. However, these conclusions must be verified by a long-term evaluation study with a larger sample size.

## Abbreviations

DSA: digital subtraction angiography
ENBD: endoscopic nasal biliary drainage
EPBD: endoscopic papillary balloon dilation
EPT: endoscopic pancreatic sphincterotomy
ERCP: endoscopic retrograde cholangiopancreatography
EST: endoscopic sphincterotomy
PEP: post- endoscopic retrograde cholangiopancreatography pancreatitis
PS: pancreatic stent
SOD: sphincter of oddi dysfunction
